# Patients with seronegative rheumatoid arthritis have a different phenotype than seropositive patients: A clinical and ultrasound study

**DOI:** 10.3389/fmed.2022.978351

**Published:** 2022-08-16

**Authors:** Natalia Carbonell-Bobadilla, Carina Soto-Fajardo, Luis M. Amezcua-Guerra, Ana Beatriz Batres-Marroquín, Tania Vargas, Adrian Hernández-Diazcouder, Valentin Jiménez-Rojas, Ana Cristina Medina-García, Carlos Pineda, Luis H. Silveira

**Affiliations:** ^1^Department of Rheumatology, Instituto Nacional de Cardiología Ignacio Chávez, Mexico City, Mexico; ^2^Rheumatology Division, Instituto Nacional de Rehabilitación Luis Guillermo Ibarra Ibarra, Mexico City, Mexico; ^3^Department of Immunology, Instituto Nacional de Cardiología Ignacio Chávez, Mexico City, Mexico; ^4^Department of Health Care, Universidad Autónoma Metropolitana-Xochimilco, Mexico City, Mexico

**Keywords:** rheumatoid arthritis, ultrasound, rheumatoid factor, anti-citrullinated protein antibodies, cardiovascular risk, seronegative rheumatoid arthritis

## Abstract

**Introduction:**

Rheumatoid arthritis (RA) is an inflammatory disease whose clinical phenotype largely depends on the presence of rheumatoid factor (RF) and anti-citrullinated protein antibodies (ACPA). Seronegative RA appears to be a less severe disease, but this remains controversial. This study aimed to assess whether seronegative patients show a less severe disease than seropositive patients.

**Methods:**

A cross-sectional study was conducted on RA outpatients from a single center. Clinical activity scales, laboratory evaluations, and cardiovascular risk scores were assessed. Musculoskeletal ultrasound (US) examinations were performed.

**Results:**

One hundred and fourteen patients were enrolled. Eighty-five were seropositive (76% women) and 29 seronegative (93% women). Seropositive patients had a younger age at disease onset (43 ± 14 vs. 54 ± 11; *p* = 0.001) and used sulfasalazine (47 vs. 17%; *p* = 0.004) and glucocorticoids (36 vs. 10%; *p* = 0.007) more frequently. No differences in clinical activity scales and in 10-year cardiovascular risk were observed. Pathological US data were found more frequently in seropositive patients in the 2^nd^ metacarpophalangeal (MCP) joint, both in grayscale (71 vs. 38%; *p* = 0.008) and in power Doppler (PD; 53 vs. 9%; *p* < 0.001); erosions (36 vs. 9%; *p* = 0.020) were also more frequent. We found greater severity of PD signals in the 2nd MCP and 3rd MCP joints of the seropositive patients, while synovitis severity was higher only in the 2nd MCP joints. The percentage of total joints with erosions (9 vs. 1%; *p* < 0.001) and 2nd MCP joints with erosions (25 vs. 7%; *p* < 0.001) was higher in seropositive patients.

**Conclusion:**

RA patients show a differentiated phenotype according to their ACPA and RF status. In seronegative patients, RA begins later in life and has a lower requirement for antirheumatic therapies. On US evaluation, seropositive patients show more joint damage, especially in MCP joints. Despite this, long-term cardiovascular risk is similar among RA patients, regardless of their RF and ACPA status.

## Introduction

Rheumatoid arthritis (RA) is a chronic disease that affects ~1% of the population. In Mexico, the prevalence has been estimated at 1.6%, according to the COPCORD methodology (1). RA is classified according to seropositivity for rheumatoid factor (RF) and anti-citrullinated protein antibodies (ACPA). Although seronegative RA (SNRA) appears to be less severe in its presentation and clinical course than seropositive RA (SPRA), there are still controversies because there are studies in which these differences do not exist. Furthermore, 20–30% of RA patients do not have ACPA and RF, and erosive RA can occur without these antibodies. Mouterde et al. in 2019 described the disease course of patients without RF or ACPA in an inception cohort of patients with early inflammatory arthritis. This ESPOR cohort included 748 patients and showed that, at a 3-year follow-up, SNRA patients had mean disease activity and quality of life similar to that observed in SPRA patients; additionally, the proportion of patients who achieved disease remission was similar. Despite this, the modified total Sharp score and radiographic progression at 3 years were lower in the SNRA group. These patients also used less conventional and biologic disease-modifying antirheumatic drugs (DMARDs) and glucocorticoids than seropositive patients (2).

It is currently unclear whether SPRA patients have a worse disease course than SNRA patients on measures of disease activity and radiological outcomes. Studies have reported increased disease severity and impaired function in patients with SPRA, both at disease presentation and after DMARD treatment (3). In contrast, other studies reported that SNRA patients had more severe inflammatory activity than SPRA as assessed by ultrasound (US) and plain radiography (4, 5). These discrepancies may be attributed to differences in the patient populations studied, inclusion criteria, and measures of disease activity between different studies. Choi S et al. demonstrated that patients with SNRA manifested more active disease at presentation, with a better response to DMARD treatment than patients with SPRA (6).

Compared to the general population, a considerably higher risk of cardiovascular disease (CVD) is observed in RA (7). Dyslipidemia, diabetes, a family history of CVD, and elevated body mass index are the associated risk factors in RA patients (8). Data suggest that RA-related factors, such as sustained inflammation, are also associated with increased risk in these patients (9). CVD mortality has been associated with the level of inflammation, the HLA-DRB1^*^0404 allele (10), the use of glucocorticoids (11), and the presence of characteristic RA antibodies (12). High C-reactive protein (CRP) levels among RA patients correlate with lower levels of total cholesterol, LDL-C, and HDL-C; at the same time, elevated CRP is associated with an increased CVD risk (13). Several algorithms that quantify CVD risk are available for use in the general population, which also apply to RA patients. These calculators use traditional parameters such as age, gender, blood pressure, smoking, cholesterol levels, and diabetes to calculate CVD risk (14). Risk prediction models provide a valuable starting point to initiate the primary prevention of CVD risk.

This study aimed to assess whether SNRA patients have a clinical and ultrasonographic less severe disease than SPRA patients.

## Materials and methods

### Patients

Observational, cross-sectional study that included consecutive outpatients diagnosed with RA according to the 2010 American College of Rheumatology/European League Against Rheumatism (ACR/EULAR) (15) classification criteria who attended the rheumatology clinic of a single tertiary care hospital. Patients were divided into two groups according to their antibody status. Patients with overlap syndrome, malignant neoplasms, hepatitis B or C virus infection, HIV, other active infections, or who had received rituximab in the last year were excluded.

The local ethics committee approved the protocol. The study was performed following the Declaration of Helsinki (16). Patients consented to participate, authorizing the use of clinical, laboratory, and imaging data for research purposes.

### Clinical and laboratory assessments

All individuals underwent a detailed evaluation, including medical history, musculoskeletal examination, ultrasound evaluation and laboratory tests. Patients were classified as SPRA if they had positive RF or ACPA and SNRA if both antibodies were negative. In addition, sociodemographic variables, age at onset of joint symptoms, age at diagnosis, smoking habit, and body mass index were recorded. Laboratory data were also collected, including platelets, leukocytes, CRP, and erythrocyte sedimentation rate. Finally, pharmacological therapies at the time of study enrollment were recorded. The extent of disease activity was evaluated using the Disease Activity Score-28 (DAS28-CRP), the Clinical Disease Activity Index (CDAI), and the Simplified Disease Activity Index (SDAI).

We calculated the ten-year cardiovascular risk using the QRISK®3-2018, the Framingham Risk Score, the Reynolds Risk Score, and the 2013 Atherosclerotic Cardiovascular Disease (ASCVD) Risk Estimator in the respective online calculators.

### Ultrasound assessment

Musculoskeletal US (MSUS) examinations were performed using the MyLab^TM^X7 system (Esaote Biomedica, Genoa, Italy) equipped with a 6 to 18 MHz broadband linear transducer. Two rheumatologists (CP and CSF) trained in MSUS, who were blinded to the clinical and laboratory data, scanned all patients. Bilateral MSUS examination was performed of the wrists, 2nd and 3rd metacarpophalangeal (MCP) joints, elbow (anterior and posterior joint recess), knee (suprapatellar and lateral parapatellar joint recess) and ankle (anterior recess of the ankle joint, and peroneal and tibialis posterior tendons) (17).

All US examinations were performed using a multiplanar technique following the EULAR guidelines (18). Assessment of inflammation and neovascularity in joints and tendons was accomplished by Power Doppler (PD) with a pulse repetition frequency of 750 kHz and a Doppler frequency between 6–8 MHz. Special attention was paid to avoiding unnecessary probe pressure and maintaining the relaxation of tendons.

### Ultrasound interpretation

We used current OMERACT (Outcome Measures in Rheumatology) definitions for ultrasonographic pathology and elementary lesions for rheumatic disorders (19). In addition, images were scored for synovitis on grayscale (GS) and PD according to the EULAR-OMERACT scoring system, which divides the severity of synovitis and intensity of PD signals from normal (grade 0) to severe (grade 3) (20). An overall GS and PD signal score was calculated as the sum of GS synovitis, PD synovitis and GS tenosynovitis and PD tenosynovitis with the range of scores of 0–36 for GS synovitis, 0–36 for PD synovitis, 0–12 for GS tenosynovitis and 0–12 for PD tenosynovitis.

Finally, in our study, the definition of disease severity is based on the concepts of disease activity using activity indexes (DAS28-CRP, CDAI, SDAI), cardiovascular risk and ultrasonographic evaluation (synovitis in GS and PD, and structural damage/erosions).

### Statistical analysis

Discrete variables were described using proportions and percentages, and differences were evaluated using the chi-square test. Means ± standard deviation (SD) or medians with interquartile range (IQR) were used to describe continuous variables, and differences were evaluated using the Student's *t*-test or the Mann–Whitney *U*-test, respectively.

To assess the association between the status of seropositivity and joint damage (bone erosions), linear regression was performed with the total of bone erosions as the dependent variable and the status of seropositivity as the independent variable. Similarly, to assess the association between the status of seropositivity and synovitis in GS and PD, linear regression was performed with the synovitis in the 2nd MCP joint as the dependent variable and the status of seropositivity as the independent variable.

Analyzes were two-tailed, and a *p* < 0.05 value was set for significance. The Graph Pad Prism version 9.3.1 software (Graph Pad Inc, La Jolla, CA, USA) was used for the calculations.

## Results

Between July 1, 2019, and May 28, 2022, 114 patients with RA were enrolled (Table 1, Supplementary Table 1). Eighty-five patients were SPRA (76% female), and 29 were SNRA (93% female). SNRA patients had an older age at the onset of the disease (54 ± 11 years vs. 43 ± 14 years; *p* < 0.001), although a similar duration of the disease, so the average age at recruitment was also significantly older (63 ± 9 years vs. 54 ± 13 years; *p* < 0.001). In contrast, the frequency of diabetes, overweight and obesity, hypertension, and history of coronary artery disease was similar. We also found no differences in the degree of disease activity or DMARDs use, except for greater use of sulfasalazine in SPRA patients (47% vs. 17; *p* = 0.004). Regarding the doses of DMARDs, SPRA patients used higher doses of methotrexate, both in the total population and in the subgroup of patients with US [17.5 (IQR 15–23.12) vs 13.75 (IQR 7.5–17.5), *p* = 0.003 and 17.5 (IQR 15–25) vs 15 (IQR 7.5–17.5), *p* = 0.006]. The frequency of use (36 vs. 10%; *p* = 0.007) and the average prednisone dose (7.5 mg/day vs. 2.5 mg/day; *p* = 0.033) was higher in SPRA patients.

**Table 1:** Clinical and laboratory features of patients with rheumatoid arthritis.

	**Seropositive patients** **(*n* = 85)**	**Seronegative patients** **(*n* = 29)**	***p***
Age, years	54 ± 13	63 ± 9	**<0.001**
Female, *n* (%)	65 (76)	27 (93)	0.050
Age of disease onset, years	43 ± 14	54 ± 11	**<0.001**
Disease duration, years	3.7 ± 5.0	3.1 ± 3.6	0.550
BMI, kg/m^2^	26.8 ± 4.7	26.3 ± 4.4	0.631
Smoking, *n* (%)	8 (9)	1 (3)	0.303
Diabetes, *n* (%)	23 (27)	9 (31)	0.680
Hypertension, *n* (%)	24 (28)	13 (44)	0.099
CAD, *n* (%)	9 (10)	2 (6)	0.561
*Disease activity*			
•DAS28-CRP, median (IQR)	2.9 (2.1–3.7)	2.5 (1.7–3.5)	0.199
•SDAI, median (IQR)	12.6 (6.7–22.8)	10.6 (3.5–20.0)	0.363
•CDAI, median (IQR)	9 (4–17)	8 (3–18)	0.383
•Extra-articular manifestations, n (%)	9 (10)	2 (6)	0.561
*Drug therapies, n (%)*			
•Methotrexate	66 (77)	26 (89)	0.157
•Sulfasalazine	40 (47)	5 (17)	**0.004**
•Leflunomide	15 (17)	4 (13)	0.630
•Hydroxychloroquine	47 (55)	14 (48)	0.512
•Statins	12 (14)	7 (24)	0.211
•PDN	31 (36)	3 (10)	**0.007**
•PDN dose, mg/day, median (IQR)	7.5 (5–10)	2.5 (2.5–3.75)	**0.033**
*Laboratory studies*			
•WBC, 1x10^3^ per mm^3^	6.9 ± 2.0	5.8 ± 1.5	**0.009**
•Neutrophils, 1x10^3^ per mm^3^	4.4 ± 1.8	3.5 ± 1.2	**0.011**
•Lymphocytes, 1x10^3^ per mm^3^	1.6 ± 0.5	1.6 ± 0.4	0.994
•NLR	2.9 ± 1.7	2.1 ± 0.8	**0.036**
•Hemoglobin, g/dl	13.8 ± 1.6	13.4 ± 1.7	0.348
•Platelets, 1x10^3^ per mm^3^	277 ± 81	279 ± 116	0.923
•Glucose, mg/dl	98.7 ± 28.9	98.8 ± 17.3	0.981
•Creatinine, mg/dl	0.74 ± 0.23	0.75 ± 0.19	0.791
•Albumin, g/dl	4.1 ± 0.2	4.1 ± 0.2	0.967
•Cholesterol, mg/dl	168 ± 34	177 ± 34	0.232
•HDL-C, mg/dl	49 ± 13	53 ± 12	0.160
•Triglycerides, mg/dl	137 ± 74	129 ± 48	0.304
•ESR, mm/h	20.7 ± 17.9	19.6 ± 16.5	0.790
•hs-CRP, mg/L	11.4 ± 15.1	6.7 ± 9.1	0.118

*Data are presented as mean ± standard deviation unless otherwise specified. Significant p-values are in bold*.

*BMI, body mass index; CAD, coronary artery disease; CDAI, Clinical Disease Activity Index; DAS28-CRP, Disease Activity Score 28-joint counts; ESR; erythrocyte sedimentation rate; HDL-C, high-density lipoprotein cholesterol; hs-CRP, high-sensitivity C-reactive protein; NLR; neutrophil-to-lymphocyte ratio; PDN, prednisone; SDAI; Simple Disease Activity Index; WBC, white blood cells*.

### Clinical assessment

The extent of disease activity and extra-articular manifestations were similar in both study groups (Table 1). Laboratory studies showed that SPRA patients had higher leukocyte (6.9 ± 2.0 x10^3^ vs. 5.8 ± 1.5 x10^3^; *p* = 0.009) and neutrophil (4.4 ± 1.8 x10^3^ vs. 3.5 ± 1.2 x10^3^; *p* = 0.011) counts, resulting in a higher neutrophil/lymphocyte ratio (2.9 ± 1.7 vs. 2.1 ± 0.8; *p* = 0.036). Meanwhile, blood cells other than leukocytes, glucose, creatinine, albumin, and lipids were similar between patients. No differences were observed in acute-phase proteins.

### Cardiovascular risk scores

Table 2 summarizes the risk of coronary artery disease or stroke at 10 years. There is a notable trend for SNRA patients to be at higher risk than SPRA patients, although none of these differences reached statistical significance. Cardiovascular risk measured by the different scales was only correlated with CRP values (Spearman's rho: QRISK®3-2018, 0.53, *p* < 0.001; Framingham Risk Score, 0.4, *p* = 0.001; Reynolds Risk Score, 0.53, *p* < 0.001; and ASCVD Risk Estimator, 0.52, *p* < 0.001), while the rest of the variables, including synovitis in GS and PD, ACPA and RF, among others, did not correlate with cardiovascular risk.

**Table 2:** Risk of coronary heart disease or stroke at 10 years.

	**Seropositive patients** **(*n* = 85)**	**Seronegative patients** **(*n* = 29)**	***p***
QRISK®3–2018	8.0 (1.8–20.5)	9.6 (5.5–20.1)	0.122
Framingham risk score	4 (2–10)	4 (3–6)	0.468
Reynolds risk score	1.7 (0.6–4.5)	2.5 (1.1–4.6)	0.303
ASCVD risk estimator	2.7 (0.6–10.1)	4.6 (2.0–9.7)	0.124

*Data are presented as median (interquartile range)*.

*ASCVD, Atherosclerotic Cardiovascular Disease*.

### US assessment

Musculoskeletal US was performed in 49 SPRA patients and 21 with SNRA. A total of 12 joints per patient were evaluated, namely elbows, wrists, 2nd and 3rd MCP joints, knees, and ankles, in addition to peroneal and tibialis posterior tendons. Table 3, Supplementary Table 2 summarizes the main joint findings. US joint inflammation was found more frequently in SPRA patients in the 2nd MCP joint, both in GS (71 vs. 38%; *p* = 0.008) and PD (53 vs. 9%; *p* < 0.001) assessments; structural joint damage manifested as bone erosions (36 vs. 9%; *p* = 0.020) were also more frequent. In contrast, no significant differences were observed in the frequency of pathologic findings in any of the other joint areas, despite a persistent trend toward greater damage in SPRA patients. We found no differences in the involvement of the posterior tibial and peroneal tendons between groups.

**Table 3:** Pathological findings observed on ultrasound in 12 main joint areas.

	**Seropositive patients** **(*n* = 49)**	**Seronegative patients** **(*n* = 21)**	***p***
2nd MCP joint			
· Grayscale	35 (71)	8 (38)	**0.008**
· Power Doppler	26 (53)	2 (9)	<**0.001**
· Erosions	18 (36)	2 (9)	**0.020**
3rd MCP joint			
· Grayscale	30 (61)	10 (47)	0.291
· Power Doppler	21 (42)	4 (19)	0.056
· Erosions	4 (8)	0	0.138
Wrist			
· Grayscale	36 (73)	14 (66)	0.563
· Power Doppler	29 (59)	9 (42)	0.208
· Erosions	10 (20)	1 (4)	0.099
Elbow			
· Grayscale	25 (51)	10 (47)	0.794
· Power Doppler	5 (10)	1 (4)	0.456
· Erosions	5 (10)	0	0.128
Knee			
· Grayscale	28 (57)	16 (76)	0.130
· Power Doppler	9 (18)	8 (38)	0.077
· Erosions	1 (2)	0	0.509
Ankle			
· Grayscale	23 (46)	10 (47)	0.958
· Power Doppler	10 (20)	2 (9)	0.268
· Erosions	3 (6)	0	0.246

*MCP, metacarpophalangeal joint. Significant p-values are in bold*.

**Figure 1:**
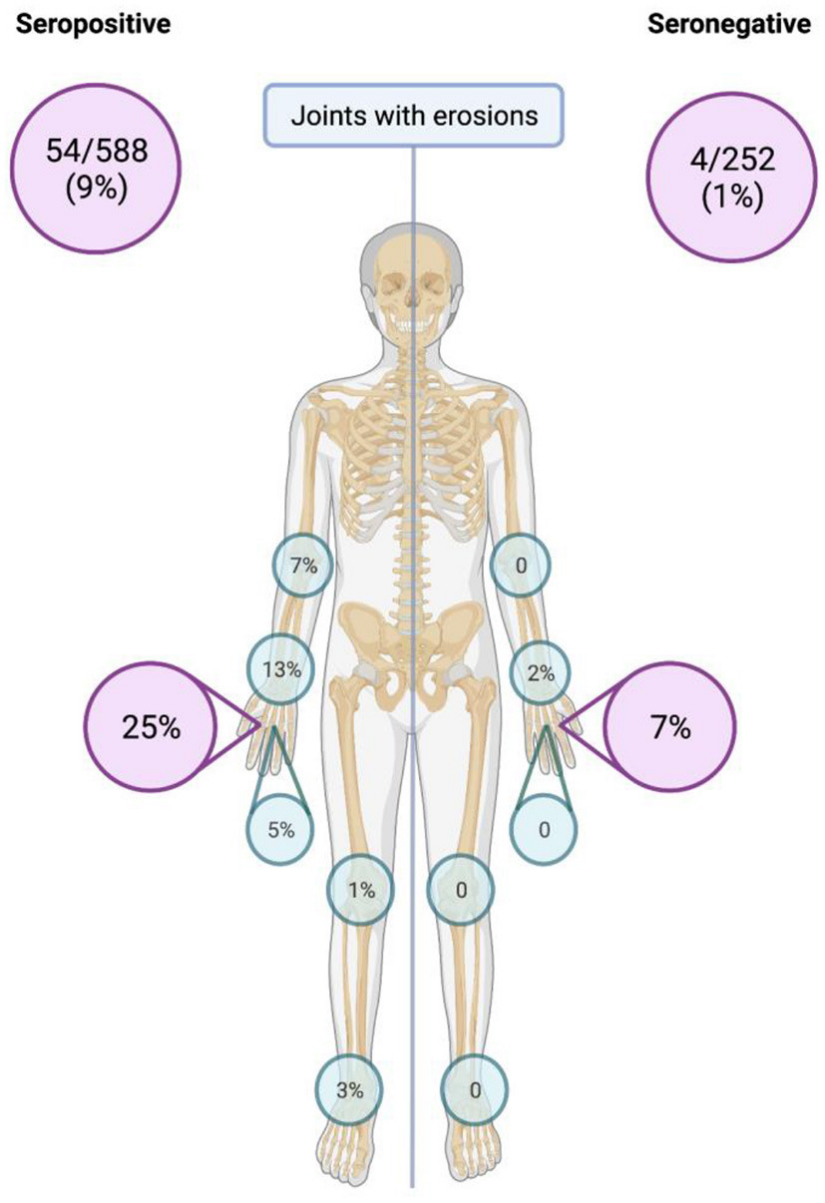
Total number and percentage of joints with structural joint damage (erosions) detected by ultrasound.

Subsequently, we analyzed the US findings according to the severity of the elementary lesions in the small joints of the hands (Table 4). We found greater severity in the PD signals in the 2nd MCP (median 0, IQR 0–0.25 vs. 0, 0–0; *p* < 0.001) and 3rd MCP (0, 0–0 vs. 0, 0–0; *p* = 0.011) joints of the SPRA patients, while the GS was higher only in the 2nd MCP joint (0, 0–2 vs. 0, 0–0; *p* < 0.001). There were no differences in the wrists. The percentage of total joints displaying evidence of structural damage (erosive disease) (9 vs. 1%; *p* < 0.001) and 2nd MCP joints with erosions (25 vs. 7%; *p* < 0.001) was significantly higher in SPRA patients (Figure 1).

**Table 4:** Ultrasonographic findings, according to the severity of the lesions, in hand joints.

	**Seropositive patients** **(*n* = 49)**	**Seronegative patients** **(*n* = 21)**	***p***
2^nd^ MCP joints			
· Grayscale	0 (0–2)	0 (0–0)	**<0.001**
· Power Doppler	0 (0–0.25)	0 (0–0)	**<0.001**
3^rd^ MCP joints			
· Grayscale	0 (0–1)	0 (0–0.25)	0.058
· Power Doppler	0 (0–0)	0 (0–0)	**0.011**
Wrists			
· Grayscale	1 (0–2)	1 (0–1)	0.116
· Power Doppler	0 (0–1)	0 (0–1)	0.142

*Data are presented as median (interquartile range). Significant p-values are in bold*.

In multivariate analysis, only seropositivity status and disease of duration were associated with bone erosions (damage) (Table 5). For synovitis, differences were only found in the 2nd MCP, while seropositivity status and DAS-28 CRP score were associated with synovitis in this joint, both in GS and PD (Supplementary Table 3). In the multivariate analysis for erosions and synovitis, we included other variables such as gender, drugs used, and HAQ, but they were not significant.

**Table 5:** Results of linear regression analyses of seropositivity status in relation to the joint damage (bone erosions).

**Independent variable**	**Beta (95% CI)**	***p*-value**
Univariable analysis		
Constant	0.2 (−0.35 to 0.75)	0.4
Status of seropositivity	0.29 (0.16 to 1.47)	0.015
Multivariable analysis		
Constant	−0.10 (−0.7 to 0.5)	0.7
Status of seropositivity	0.28 (0.14 to 1.42)	0.016
Disease duration	0.24 (0.003–0.082)	0.03

*CI, Confidence Interval*.

## Discussion

SNRA is considered a less severe disease than SPRA, although, controversy still exists (2, 4, 5). In this study, we aimed to analyze the differences in SNRA as a less severe disease compared to SPRA.

We found significant differences in the age of onset of the disease since seronegative patients were older (54 ± 11 years), compared to seropositive patients (43 ± 14 years). No differences in extra-articular manifestations and the clinical activity scales were observed.

There are some similarities and some discrepancies in the literature with our findings. Mouterde et al. aimed to describe the disease course of patients without RF and ACPA in an inception cohort of early inflammatory arthritis patients and to determine baseline predictors of fulfilling 2010 ACR/EULAR criteria for RA within 3 years. They used a large, prospective, early-arthritis cohort from the community. The disease was less active based on DAS28-ESR and also less severe in terms of the functional index and radiographic score at baseline in the seronegative patients than in the seropositive patients (21). These results agree with those of the Norfolk Arthritis Register (NOAR) (3), but not with the Canadian early-arthritis cohort (CATCH), showing seronegative patients with higher mean swollen joint count, DAS28, and erosive disease, which suggests that these patients are more frequently referred to rheumatology if they have more active and severe disease. The disease progression was less severe and DMARD or glucocorticoid use less frequent in the seronegative vs. the seropositive group during follow-up in the ESPOIR cohort, which agrees with other early-arthritis cohorts (22).

Choi et al. found that SNRA patients manifested more active disease at baseline compared than SPRA patients (6). This could be partly explained by the fact that 99.5% of SPRA patients met the 2010 ACR/EULAR criteria, while only 27.5% of SNRA patients did. The 2010 ACR/EULAR criteria give much weight to serology markers to detect patients with RA early in the disease course. Therefore, seropositive patients with only one or two involved joints could be diagnosed with RA (22). This could explain the older mean age reported in our study in the SNRA group.

There were also differences in the pharmacological treatment. SNRA group needed fewer DMARDs combinations than the SPRA group, being more evident with sulfasalazine since only five of the seronegative patients was taking this drug, compared to the 47% of the SPRA group; additionally, the dose of methotrexate was higher for the SPRA group. Glucocorticoid requirement was less in the seronegative group, only three patients (10%) needed prednisone, with mean doses of 2.5 (2.5–3.75 IQR) mg per day, compared to the seropositive group with 31 patients (36%) taking prednisone with mean doses of 7.5 (5–10 IQR) mg per day (*p* = 0.007). SNRA has been considered to represent a less severe disease subset than SPRA, with less radiographic damage (23). It has been suggested that seronegative patients should be treated less aggressively than seropositive patients, which is also reflected in the 2016 EULAR treatment recommendations (24). Nordberg et al. found that in RA patients classified according to the new criteria, SNRA is not a mild form of the disease and requires intensive treat-to-target therapy similar to treatment of SPRA. In their study, there was a trend toward more radiographic damage in seronegative compared with seropositive patients, both at baseline and after 24 months. The treatment response at 3 months was better in seropositive than seronegative patients, whereas the number of patients in remission at the end of the study was similar across groups. This observation may indicate that seronegative patients might respond well to treat-to-target strategies, even if the initial treatment response is delayed compared with seropositive patients (25).

Our results differ from results presented by Choi et al. and Nordberg et al. (4, 6); however, both studies included DMARD-naive patients, and in the study by Choi et al. when following the patients they observed that those with SNRA had a better response to treatment. With these results, we could conclude that patients with SNRA during their follow-up had a lower frequency and degree of synovitis involvement.

We evaluated cardiovascular risk using scoring systems to analyze the risk of coronary heart disease or stroke at 10 years. ACPAs have been associated with coronary artery disease in a previous report (26). Some other studies have also shown that seropositive patients have more severe inflammatory activity than patients with SNRA (27). CVD mortality has been associated with sustained inflammation, the level of inflammation (9), and with the presence of characteristic RA antibodies (12). Moreover, increased risk of coronary heart disease in RA is associated with elevated CRP and erythrocyte sedimentation rate, the presence of RF and/or ACPA, as well as with highly active or severe RA (28). Rheumatoid factor and antinuclear antibodies have been associated with heart disease and overall mortality, even in patients without rheumatic diseases (29). Considering all these findings, we were expecting to find some differences between SNRA and SPRA, however, we did not find statistically significant differences in 10-year cardiovascular risk scores.

In general, MSUS results showed a higher percentage of damage and a greater degree of involvement in the right 2nd MCP joint in both GS and PD in SPRA patients. Of the rest of the joints, we found some other differences in both scales, with a greater degree of involvement in the left ankle and left 2nd MCP joint in GS and with greater frequency of PD presence in the left 2nd MCP joint and right 3rd MCP joint in the group of patients with SPRA. Only at the level of the knees did patients with SNRA have a greater degree of involvement in GS and a higher frequency of PD. With these findings, we can conclude that patients with SPRA show a tendency to have more synovitis in both GS and DP. These findings are consistent with the recently published article by Ramirez et al. in which patients with ACPA had a greater presence of proliferative synovitis (30).

Regarding erosions, recently, in the study by Grose et al. in which patients were compared according to ACPA status, they found that patients with positive ACPA had a higher proportion of erosions by US (31). Our results agree with those described in this study, in which we found a higher frequency of erosions in the SPRA group, mainly due to the higher proportion of erosions in the 2nd MCP joint, without differences in the rest of the joints; however, we believe that this is because we used a simplified score, which has the disadvantage that it does not include the joints with the highest frequency of erosions (5th MCP joint and 2nd, 3rd, and 5th metatarsophalangeal joints).

The main strength of our study is that it's one of the few studies that compare clinical, ultrasonographic, laboratory variables and cardiovascular risk between both study subgroups (SPRA vs SNRA), which allows for a better definition of the phenotype presented by patients with SNRA.

Finally, our study has several limitations. One of the main limitations is the sample size and that not all patients included in the study could undergo MSUS mainly due to complications associated with the pandemic. Another important limitation of our study is that we used a simplified score to evaluate the joints, which, although it has a very good sensitivity to evaluate synovitis in GS and PD, does not evaluate all the joints in which erosions occur more frequently. A third limitation is that we did not include the evaluation of erosions by radiographs. A final limitation is that we didn't evaluate the presence of anti-carbamylated proteins antibody.

## Conclusions

RA patients show a differentiated phenotype according to their ACPA and RF status. In seronegative patients, RA begins later in life and has a lower requirement for antirheumatic therapies. On US evaluation, seropositive patients show more joint damage, especially in MCP joints. Despite this, long-term cardiovascular risk is similar among RA patients, regardless of their RF and ACPA status.

## Data availability statement

The datasets obtained and/or analyzed during the current study will be available from the corresponding author on reasonable request.

## Ethics statement

The studies involving human participants were reviewed and approved by Ethical and Research Committee Instituto Nacional de Rehabilitation. The patients/participants provided their written informed consent to participate in this study.

## Author contributions

NC-B, CS-F, and LA-G: acquisition of data, analysis and interpretation of data, drafting of the article, critical revision of the intellectual content, and final approval of the version to be published. AB-M, TV, AH-D, VJ-R, and AM-G: acquisition of data. CP: substantial contributions to the design, acquisition of data, critical revision of the intellectual content, and final approval of the version to be published. LS: ideation of the study, substantial contributions to the design, critical revision of the intellectual content, and final approval of the version to be published. All authors contributed to the article and approved the submitted version.

## Conflict of interest

The authors declare that the research was conducted in the absence of any commercial or financial relationships that could be construed as a potential conflict of interest.

## Publisher's note

All claims expressed in this article are solely those of the authors and do not necessarily represent those of their affiliated organizations, or those of the publisher, the editors and the reviewers. Any product that may be evaluated in this article, or claim that may be made by its manufacturer, is not guaranteed or endorsed by the publisher.
