# Supplementary material for: Patients with seronegative rheumatoid arthritis have a different phenotype than seropositive patients: A clinical and ultrasound study
Source: Front Med (Lausanne). 2022 Aug 16;9:978351. doi: 10.3389/fmed.2022.978351 (PMC9424641; doi:10.3389/fmed.2022.978351)
Supplement: Supplementary file 1 [file Data_Sheet_1.docx]

Supplementary Material

# Supplementary Tables

Supplemental table 1. Clinical and laboratory features of patients with rheumatoid arthritis and musculoskeletal ultrasound.

|  | Seropositive patients  (n= 49) | Seronegative patients  (n= 21) | p |  |
| --- | --- | --- | --- | --- |
| Age, years | 54 ±12 | 62 ± 10 | 0.46 |  |
| Female, n (%) | 39 (79) | 20 (95) | 0.15 |  |
| Age of disease onset, years | 44 ± 13 | 55 ± 11 | 0.12 |  |
| Disease duration, years  median (IQR) | 5.5 (3-13) | 4 (2.5-7.5) | 0.39 |  |
| BMI, kg/m^2^ | 27 ± 4.1 | 26.4 ± 4.6 | 0.36 |  |
| Smoking, n (%) | 4 (8) | 1 (4) | 0.46 |  |
| Diabetes, n (%) | 15 (30) | 5 (23) | 0.64 |  |
| Hypertension, n (%) | 14 (28) | 10 (47) | 0.09 |  |
| CAD, n (%) | 3 (6) | 1(4) | 0.85 |  |
| *Disease activity* | | | | |
| DAS28-CRP, median (IQR) | 3.01 (2.25-3.76) | 2.9 (1.9-4) | 0.92 |  |
| SDAI, median (IQR) | 12.45 (7.6-22.1) | 12.6 (3.2-3) | 0.58 |  |
| CDAI, median (IQR) | 9.5 (5-15.7) | 9 (3-20.9) | 0.86 |  |
| Extraarticular manifestations, n (%) | 3 (6) | 2 (9) | 0.85 |  |
| *Drug therapies, n (%)* | | | | |
| Methotrexate | 40 (81) | 18 (85) | 0.73 |  |
| Dose, mg/week | 17.5 (15-25) | 15 (7.5-17.5) | **0.006** |  |
| Sulfasalazine | 20 (40) | 5 (23) | 0.21 |  |
| Dose, g/day, median (IQR) | 2 (1.62-3) | 0.4 ± 0.87 | 0.336 |  |
| Azathioprine | 2 (4) | 0 | 0.35 |  |
| Dose, g/day, median (IQR) | 150 (125) * | 0 | NC |  |
| Leflunomide | 10 (20) | 3 (14) | 0.60 |  |
| Dose, g/day, median (IQR) | 20 (17.5-20) | 20 (17.5-20) | 0.69 |  |
| Hydroxychloroquine | 27 (55) | 12 (57) | 0.99 |  |
| Dose, g/day, median (IQR) | 200 (200-200) | 200 (200-200) | 1.0 |  |
| Statins | 4 (8) | 5 (23) | 0.16 |  |
| PDN | 18 (36) | 2 (9) | **0.002** |  |
| Dose, g/day, median (IQR) | 7.5 (5-11.25) | 3.75 (2.5) * | 0.21 |  |
| Combination therapy | 33 (67) | 15 (71) | 0.70 |  |
| *Laboratory studies* | | | | |
| ·         WBC, 1x10^3^ per mm^3^ | 6.8 ± 1.8 | 5.8 ± 1.6 | 0.57 |  |
| ·         Neutrophils, 1x10^3^ per mm^3^ | 4.3 ± 1.6 | 3.4 ± 1.2 | 0.28 |  |
| Lymphocytes, 1x10^3^ per mm^3^ | 1.7 ± 0.5 | 1.7 ± 0.5 | 0.63 |  |
| NLR | 0.6 ± 0.1 | 0.58 ± 0.09 | 0.21 |  |
| Hemoglobin, g/dL | 13.8 ± 1.5 | 13.6 ± 1.8 | 0.58 |  |
| Platelets, 1x10^3^ per mm^3^ | 284 ± 83 | 274 ± 126 | **0.07** |  |
| Glucose, mg/dL | 100 ± 30.6 | 95.8 ± 10.2 | 0.48 |  |
| Creatinine, mg/dL | 0.74 ± 0.23 | 0.78 ± 0.19 | 0.31 |  |
| Albumin, g/dL | 4.2 ± 0.2 | 4.2 ± 0.2 | 0.91 |  |
| Cholesterol, mg/dL | 165 ± 30 | 175 ± 34 | 0.19 |  |
| HDL-C, mg/dL | 49 ± 14 | 55 ± 13 | 0.79 |  |
| Triglycerides, mg/dL | 135 ± 76 | 129 ± 43 | 0.27 |  |
| ESR, mm/h | 18.7 ± 16.1 | 15.9 ± 14.9 | 0.82 |  |
| hs-CRP, mg/L | 11.2 ± 16 | 7 ± 10.4 | 0.07 |  |

Data are presented as mean ± standard deviation unless otherwise specified. Significant *p* values are in bold.

*: It is impossible to calculate the 75th percentile because only two patients take this drug.

Definitions: BMI, body mass index; CAD, coronary artery disease; CDAI, Clinical Disease Activity Index; DAS28-CRP, Disease Activity Score 28-joint counts; ESR; erythrocyte sedimentation rate; HDL-C, high-density lipoprotein cholesterol; hs-CRP, high-sensitivity C-reactive protein; NLR, neutrophil-to-lymphocyte ratio; PDN, prednisone; SDAI; Simple Disease Activity Index; WBC, white blood cells; NC, cannot be calculated.

**Supplemental table 2. EULAR-OMERACT score**

|  | | | Seropositive patients  n (%) | Seronegative patients  n (%) | p |
| --- | --- | --- | --- | --- | --- |
| 2^nd^ MCP joint | GS | 0 | 14 (28.6) | 13 (61.9) | **0.03** |
|  |  | 1 | 9 (18.4) | 4 (19) |  |
|  |  | 2 | 12 (24.5) | 3 (14.3) |  |
|  |  | 3 | 14 (28.6) | 1 (4.8) |  |
|  | PD | 0 | 23 (46.9) | 19 (90.5) | **0.008** |
|  |  | 1 | 6 (12.2) | 0 |  |
|  |  | 2 | 13 (26.5) | 1 (4.8) |  |
|  |  | 3 | 7 (14.3) | 1 (4.8) |  |
| 3^rd^ MCP joint | GS | 0 | 20 (40.8) | 11 (52.4) | 0.75 |
|  |  | 1 | 11 (22.4) | 5 (23.8) |  |
|  |  | 2 | 11 (22.4) | 3 (14.3) |  |
|  |  | 3 | 7 (14.3) | 2 (9.5) |  |
|  | PD | 0 | 28 (57.1) | 17 (81) | 0.18 |
|  |  | 1 | 2 (9.5) | 10 (20.4) |  |
|  |  | 2 | 6 (12.2) | 0 |  |
|  |  | 3 | 5 (10.2) | 2 (9.5) |  |
| Wrist | GS | 0 | 13 (26.5) | 8 (38.1) | 0.26 |
|  |  | 1 | 10 (20.4) | 7 (33.3) |  |
|  |  | 2 | 18 (36.7) | 5 (23.8) |  |
|  |  | 3 | 8 (16.3) | 1 (4.8) |  |
|  | PD | 0 | 20 (40.8) | 12 (57.1) | 0.46 |
|  |  | 1 | 10 (20.4) | 4 (19) |  |
|  |  | 2 | 16 (32.7) | 5 (23.8) |  |
|  |  | 3 | 3 (6.1) | 0 |  |
| Elbow | GS | 0 | 25 (51) | 11 (52.4) | 0.94 |
|  |  | 1 | 11 (22.4) | 4 (19) |  |
|  |  | 2 | 7 (14.3) | 4 (19) |  |
|  |  | 3 | 6 (12.2) | 2 (9.5) |  |
|  | PD | 0 | 44 (89.8) | 20 (95.2) | 0.34 |
|  |  | 1 | 4 (8.2) | 0 |  |
|  |  | 2 | 1 (2) | 1 (4.8) |  |
|  |  | 3 | 0 |  |  |
| Knee | GS | 0 | 21 (42.9) | 5 (23.8) | 0.2 |
|  |  | 1 | 11 (22.4) | 3 (14.3) |  |
|  |  | 2 | 12 (24.5) | 10 (47.6) |  |
|  |  | 3 | 5 (10.2) | 3 (14.3) |  |
|  | PD | 0 | 40 (81.6) | 13 (61.9) | 0.36 |
|  |  | 1 | 3 (6.1) | 3 (14.3) |  |
|  |  | 2 | 5 (10.2) | 4 (19) |  |
|  |  | 3 | 1 (2) | 1 (4.8) |  |
| Ankle | GS | 0 | 36 (73.5) | 17 (81) | 0.10 |
|  |  | 1 | 5 (10.2) | 3 (14.3) |  |
|  |  | 2 | 8 (16.3) | 0 |  |
|  |  | 3 | 0 | 1 (4.8) |  |
|  | PD | 0 | 45 (91.8) | 21 (100) | 0.4 |
|  |  | 1 | 2 (4.1) | 0 |  |
|  |  | 2 | 2 (4.1) | 0 |  |
|  |  | 3 | 0 | 0 |  |
| Tibialis posterior tendon | GS | 0 | 33 (67.3) | 16 (76.2) | 0.77 |
|  |  | 1 | 10 (20.4) | 4 (19) |  |
|  |  | 2 | 5 (10.2) | 1 (4.8) |  |
|  |  | 3 | 1 (2) | 0 |  |
|  | PD | 0 | 43 (87.8) | 20 (95.2) | 0.7 |
|  |  | 1 | 2 (4.1) | 0 |  |
|  |  | 2 | 3 (6.1) | 1 (4.8) |  |
|  |  | 3 | 1 (2) | 0 |  |
| Peroneal tendon | GS | 0 | 41 (83.7) | 17 (81) | 0.83 |
|  |  | 1 | 6 (12.2) | 2 (9.5) |  |
|  |  | 2 | 1 (2) | 1 (4.8) |  |
|  |  | 3 | 1 (2) | 1 (4.8) |  |
|  | PD | 0 | 47 (95.9) | 19 (90.5) | 0.30 |
|  |  | 1 | 1 (2) | 2 (9.5) |  |
|  |  | 2 | 1 (2) | 0 |  |
|  |  | 3 | 0 | 0 |  |

GS, Grayscale; PD, Power-Doppler, Significant *p* values are in bold.

**Supplementary table 3. Results of linear regression analyses of seropositivity status in relation to the synovitis in GS and PD**

| **Independent variable** | | **Beta (95% CI)                    p-value** |
| --- | --- | --- |
| PD in 2^nd^ MCP | Univariable analyses  Constant  Status of seropositivity | 0.25 (-0.22 to 0.72)     0.4  0.34 (0.27 to 1.39)        0.004 |
|  | Multivariable analyses  Constant  Status of seropositivity  DAS28PCR | -0.13 (-0.64 to 0.38)      0.6  0.33 (0.28 to 1.34)         0.003  0.32 (0.12-0.58)             0.004 |
| Synovitis (GS) in 2^nd^ MCP | Univariable analyses  Constant  Status of seropositivity | 0.25 (-0.22 to 0.72)     0.4  0.34 (0.27 to 1.39)        0.004 |
|  | Multivariable analyses  Constant  Status of seropositivity  DAS28PCR | -0.13 (-0.64 to 0.38)      0.6  0.33 (0.28 to 1.34)         0.003  0.32 (0.12-0.58)             0.004 |

GS, Grayscale; PD, Power-Doppler; CI, Confidence interval
